# How could artificial intelligence aid clinicians caring for preterm neonates with respiratory pathology?

**DOI:** 10.3389/fped.2026.1885589

**Published:** 2026-10-13

**Authors:** Vithushanan Ketheeswaranathan, Surina Fordington, Caroline Hartley

**Affiliations:** Department of Paediatrics, University of Oxford, Oxford, United Kingdom

**Keywords:** apnoea, artificial intelligence, bronchopulmonary dysplasia, extubation, machine learning, premature, respiration

## Abstract

Artificial intelligence (AI) presents an opportunity to transform neonatal care. By rapidly assimilating large volumes of complex, multi-dimensional data to produce real-time predictions, machine learning (ML) models have the potential to enable personalised monitoring and treatment and earlier intervention. This narrative review aims to provide clinicians with a foundational overview of AI and its potential role in the care of preterm infants, with a particular focus on respiratory pathologies. We introduce different types of AI tools and ways to assess predictive model performance. We summarise existing literature using AI and conventional predictive models to predict respiratory conditions, including extubation readiness, bronchopulmonary dysplasia, and apnoea occurrence and risk. Finally, we discuss current limitations of AI in neonatology, barriers to clinical translation and the next steps needed to overcome them. These may include multicentre collaboration and data sharing, using dynamic inputs such as vital signs to reflect evolving physiology, and improved transparency, accessibility and training for clinicians, as well as clear guidelines for clinical application, accountability and ongoing monitoring.

## Introduction

Neonatal Intensive Care Units (NICUs) care for the sickest and most vulnerable infants in their first days of life, and precise, personalised and holistic treatment decisions are key to achieving positive outcomes. Prematurity is the most common reason for NICU admission, and preterm infants are vulnerable to numerous complications, potentially affecting multiple systems (1). With improved survival rates of preterm neonates over recent decades, owing to advances in obstetric and neonatal care, the prevalence of prematurity-associated complications is increasing (2). Respiratory pathologies are a leading cause of morbidity and mortality, attributable to risk factors including the immature physiology and morphology of preterm lungs, increased susceptibility to infection, and exposure to intensive and often prolonged respiratory support (3, 4). Respiratory complications of prematurity can be profound and have lifelong consequences, and early identification of at-risk infants is key to enabling timely intervention and improving long-term outcomes (5–7). There is a myriad of demographic, clinical and environmental factors placing preterm infants at increased risk of respiratory pathologies, making treatment decisions complex and challenging (8). In addition, preterm neonates with respiratory illness often present with subtle clinical signs and can deteriorate quickly, so swift and incisive decision-making is crucial.

Artificial intelligence (AI), and within this machine learning (ML), are emerging and rapidly growing tools within healthcare which have the potential to support clinicians with complex decision-making (9). NICUs represent an ideal environment for AI-assisted decision-making and there is growing recognition of its potential role in supporting clinicians to deliver safe, efficient and personalised care (10). The array of factors that influence a preterm infant's risk of respiratory pathology present ideal inputs for ML models, which are designed to integrate large volumes of complex information, find patterns and make predictions. Moreover, NICUs are ‘big data’ environments, with an abundance of dynamic physiological data, often continuously recorded, which can provide real-time data to train ML models (11, 12). Why, therefore, are AI tools not already incorporated into routine practice in the care of preterm infants?

In this review, we discuss the progress which has been made for AI tools in neonatal care, focusing on respiratory pathologies. We also discuss the current barriers limiting the translation of AI tools into everyday neonatal care and suggest ways of surmounting these. AI tools show promise for supporting neonatal clinicians caring for preterm infants and this review aims to provide clinicians with a basic understanding and vision of how this could be achieved.

## Methods

A narrative literature review was undertaken to summarise current research regarding AI and wider predictive tools in neonatology, specifically in relation to respiratory pathologies. Since there is significant heterogeneity in published research in this field in terms of study populations, inputs, output definitions and appraisal methodology, and our objective was to provide a broad overview of the field, a narrative review was chosen as the most appropriate synthesis method, and data was not collected systematically. Electronic searches of PubMed, Embase and Google Scholar were conducted between December 2025 and August 2026 using combinations of the following search terms: ‘neonate’, ‘preterm’, ‘infant’, ‘NICU’, ‘artificial intelligence’, ‘AI’, ‘machine learning’, ‘predictive model’, ‘extubation’, ‘bronchopulmonary dysplasia’, ‘apnoea’ and ‘sepsis’. Reference lists of relevant articles were also screened to identify additional publications. Original studies, including preprints, involving the development and appraisal of novel ML and deep learning models were prioritised for inclusion and review articles were also used to provide background and contextual information. The most relevant studies involving the development and appraisal of conventional statistical prediction models (e.g. logistic regression) and diagnostic accuracy studies, which the authors deemed were useful for building readers’ understanding and providing context, were also included.

## What is artificial intelligence?

AI is a term describing computational systems that perform tasks associated with human intelligence (13). ML is a subfield of AI where previously observed data is analysed to build and ‘train’ models. ML models are then ‘tested’ on a separate data (sub)set, to evaluate how effective they are at predicting outcomes, based on patterns learned from the training data (14). Conventional statistical models, such as logistic regression models, are distinct from ML models, and aim to explain relationships between specific variables of interest and estimate effects on an outcome in question. In contrast, ML models make fewer assumptions about the underlying data and instead aim to maximise prediction accuracy rather than enabling inferences about variables. Many neonatal prediction models in the literature are in fact conventional statistical models rather than true AI tools. Although both conventional statistical models and ML models can be integrated into clinical decision support systems (these are software systems that incorporate predictive models or diagnostic tools into clinically-useful workflows), it cannot be assumed that a clinical support tool or calculator automatically stems from AI. Nevertheless, conventional statistical models often inform ML models, so this review will include some selected conventional statistical models, since they provide useful context to the evolution and current status of overall predictive model development.

Currently, ML models learn patterns from the training dataset via three main approaches: a) supervised b) unsupervised and c) reinforcement learning (15). In supervised learning, a model is trained on labelled data, then tested on a separate set of data with labels withheld to determine how accurately the model can predict these unseen labels (14). It is the most common mode of ML used within clinical practice (e.g. for disease diagnosis) and can include classification (predicting categories) or regression (predicting continuous values). An example in respiratory neonatology would be a model predicting bronchopulmonary dysplasia (BPD), which is trained on data labelled according to whether the infant developed clinically-determined BPD or not. The model would be tested by predicting unseen labels on test data, with accuracy of the model assessed by comparing the predicted label with the true label (16). If the model were to achieve high test accuracy, it could be used to predict whether a future infant will develop BPD; identified high-risk infants could then, for example, receive targeted interventions to mitigate risk of BPD development, as will be discussed later (17).

In unsupervised learning, a model is trained by identifying latent patterns or groups within an unlabelled dataset, for example, in the identification of a new disease subtype. It is particularly useful for heterogenous and sizeable data, and has shown promise identifying genetic biomarkers for disease, for example (18, 19). With so many remaining unknowns regarding neonatal physiology and development, unsupervised models, which can identify new relationships in complex data sets, have utility. Hybrid approaches combine supervised and unsupervised learning, such as ‘one-class’ or ‘semi-supervised’ approaches: here, models are trained exclusively on one ‘class’ (label) then tested on a dataset to identify data that lies outside of this ‘class’, thus identifying potential anomalies. Within neonatology, this type of modelling has been used to predict low birthweight, for example (20).

In reinforcement learning, a computer agent learns to maximise reward by repeated trial and error interactions with its environment (15, 21). This type of ML has hitherto had limited use in clinical practice. However, retrospective studies show potential use in optimising mechanical ventilation for critically ill adults through a reward system based on survival rates and in the development of dynamic treatment regimens (21, 22).

Finally, deep learning is a subset of ML that is rapidly evolving within healthcare (23, 24). Its functioning is derived from neural networks within the human brain that process and interpret information (25). In deep learning, artificial neural networks are presented as layers with interconnections known as ‘synapses’; ‘synaptic weights’ are coefficients that modify the strength of these connections, thus assigning significance to the data patterns these artificial neuronal networks present (24). As the layers increase, more complex relationships between inputs and outputs can be modelled. Deep learning models can be either supervised, unsupervised or semi-supervised. Importantly, deep learning models autonomously extract features from datasets, rather than relying on extraction methods devised by humans seen in more conventional models (25, 26), potentially increasing their predictive ability. Deep learning ML models show promise in neonatology, and have been used, for example, for integrating video and physiological data to predict the impact of care manipulation, and in assessing brain maturity (27–30).

### How can ML model performance be assessed and what are the common challenges?

The clinical utility of AI tools depends on their ability to produce reliable predictions. This section summarises key metrics used to appraise ML models’ predictive performance, which will be referenced throughout this review. Model performance can either be tested on a test set which is fully independent from the training set, or using a process known as cross-validation, where the training set is split into sub-sets and the model repeatedly trained and tested on separate sub-sets.

To assess models producing binary classifications (such as presence or absence of a disease), metrics include sensitivity (percentage of true positives correctly identified), specificity (percentage of true negatives correctly identified), positive predictive value (percentage of positively classified subjects which are truly positive) and negative predictive value (percentage of negatively classified subjects which are truly negative) (31, 32). Ensuring adequate sensitivity and specificity is key to avoiding under- or overtreatment resulting from model use, but the most appropriate weighting of each metric depends on the exact clinical question (31). For instance, in a model predicting infants at risk of having sepsis, if a positive result triggers clinician review, then false negatives could be considered more detrimental than false positives; however, if a positive prediction triggers an invasive procedure such as lumbar puncture, then minimising false positives (ensuring high specificity) becomes more significant. To summarise these metrics, ‘accuracy’ describes the fraction of samples which are correctly identified. However, caution is needed if there are unequal numbers in each outcome group, known as class imbalance. Class imbalance is especially prevalent in neonatology, since true disease cases are usually much less prevalent than healthy cases. To ensure accurate assessment of model performance, balanced accuracy (the average sensitivity and specificity) should be used when class imbalance occurs. Figure 1 summarises this concept and some of the other key challenges and pitfalls in ML model development and evaluation, with a focus on neonatal research.

**Figure 1:**
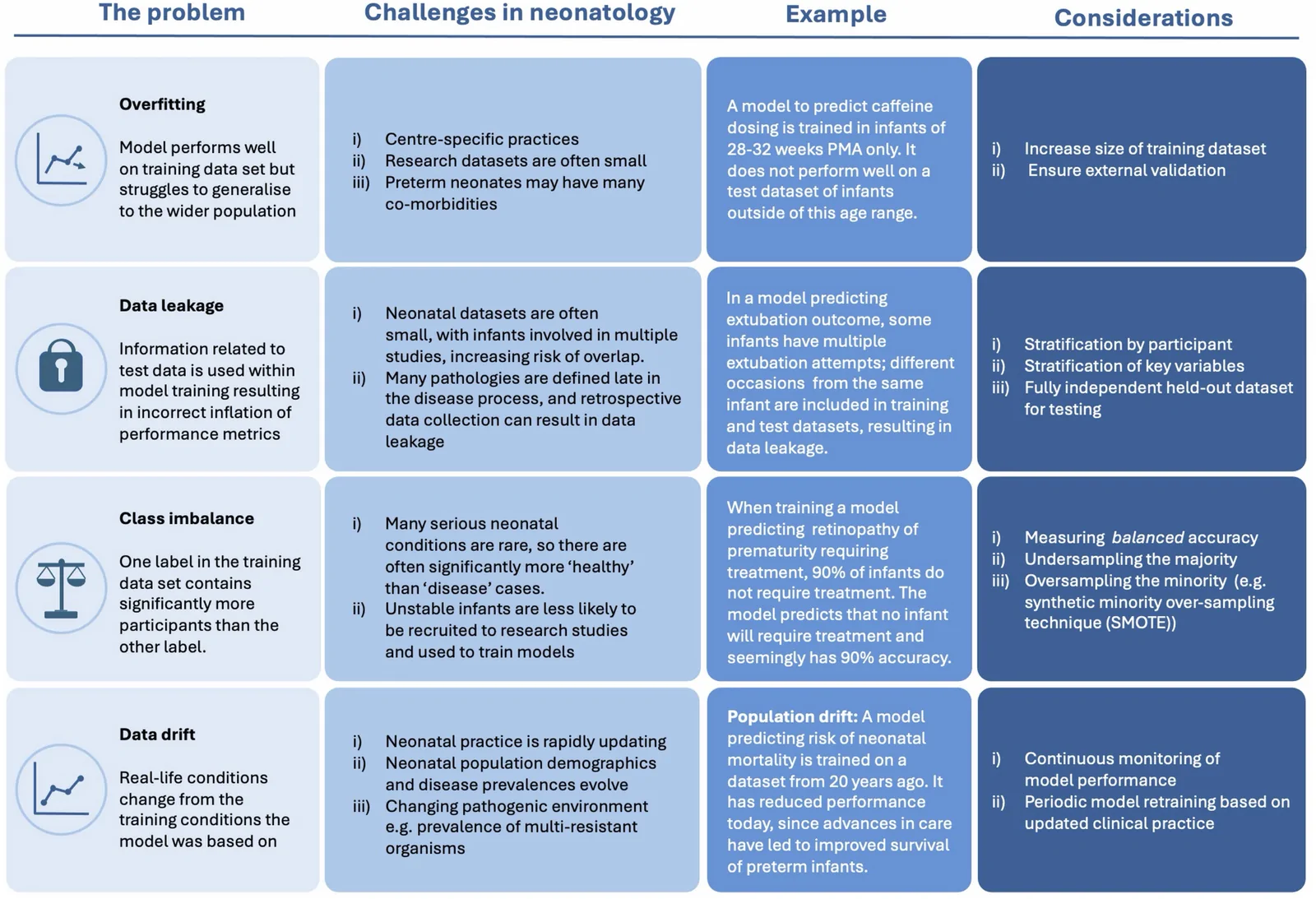
Key challenges of developing machine learning (ML) models in neonatology.

Whilst many ML models make predictions in the form of class labels, in practice the underlying model often produces a continuous output (such as a probability that the data point belongs to one class or the other) to which a threshold is applied to determine the class label. The area under the receiver operating characteristics curve (AUROC) can be used to evaluate a classifier's performance at all possible thresholds; an AUROC value of 1 indicates perfect classification, whereas 0.5 indicates random classification (33). Similarly, precision-recall (PR) curves examine the relationship between positive predictive value and sensitivity, with 1 corresponding to perfect classification (34). Moreover, calibration can be used to assess how closely a model's predicted probabilities match actual observed outcomes. For example, a well-calibrated clinical model would predict that 25% of infants would develop a pathology, out of all infants assigned a risk score of 25%. The Brier score is a single number that reflects the discrepancy between probabilistic predictions and observed outcomes. It involves calculating the mean squared difference between the two, with lower values indicating smaller prediction errors (35). Decision curve analysis enables clinicians to assess the utility of the model in clinical practice, rather than solely looking at its statistical accuracy, by assessing how the model's net benefit at guiding treatment decisions changes with different thresholds and comparing this with the net benefit of treating all patients (treat-all) or treating no patients (treat-none) (36, 37). Finally, Youden's index is an objective approach to identify which threshold maximises the sum of the sensitivity and specificity of a model and is often used as a summary measure of the receiver operating characteristic curve. However, this method equally weights false positives and false negatives which is rarely true in clinical practice (38). Many other metrics of model performance can also be used, depending on the assessment aim and data distribution. See Simon and Aliferis’ book for a more comprehensive overview of ML development, assessment and common pitfalls (32).

In addition to assessing the initial model, a key consideration for clinical use is how well the model translates to a more general clinical setting. Ideally, a model would be tested in real-life settings, reflecting population variations in infant physiology, demographics and treatment protocols. In the research setting, a more feasible initial method can be to internally validate a model on a separate, similar set of infants, whose data have been gathered using similar techniques, but are kept separate from the training dataset. However, training a model on a specific population runs the risk of overfitting, whereby a model performs well on its specific training cohort, but struggles to be applied to a wider population (see Figure 1). Often, this is because the training dataset does not represent the wider population, especially if trained in a single hospital, as practice may be protocol-driven and centre/clinician-specific. To ensure generalisability, testing the model on a completely separate population, known as external validation, is essential (39). These concerns come under the concept of fairness, which is a principle that evaluates models based on their ability to perform equally across patient subgroups. A model may perform significantly worse when evaluated specifically within underrepresented ethnic, sex or gestational age subgroups, thus amplifying existing health disparities seen in clinical practice. Models should therefore routinely be evaluated across various under-represented subgroups to ensure comparable performance when compared to that observed with the overall population, prior to being incorporated into clinical practice (40). Alongside external validation, temporal validation is a valuable performance assessment tool. It involves testing a model's performance on more recent data from the same centre as the original training dataset, thus evaluating whether performance is maintained over time as changes occur such as in clinical practice, equipment and case-mix (41).

Frameworks have been established for both researchers and reviewers to ensure consistent reporting and evaluation of prediction models. Specifically, TRIPOD + AI is a framework for researchers which outlines the minimum information that should be reported (42). PROBAST + AI is a framework for readers and reviewers to evaluate the risk of bias and applicability of prediction models. The use of these frameworks for clinical prediction models will improve reproducibility and encourage consistent evaluation of future studies (43).

## Using ML to predict respiratory-related outcomes in neonatology

Predictive models have been applied to multiple preterm respiratory pathologies, including prediction of extubation readiness, bronchopulmonary dysplasia (BPD) development, and the risk of apnoea and apnoea-related instability. This next section will outline some key ML and deep learning models, as well as relevant conventional statistical models, which have shaped understanding in this field, and may play a role in clinical care in the future.

### Predicting extubation outcomes

Endotracheal intubation and mechanical ventilation can be life-saving interventions for many preterm infants. However, prolonged mechanical ventilation can increase volutrauma/barotrauma, ventilator-associated infections and development of BPD, so current practice aims to extubate early to mitigate potential complications (44). Extubating too early, however, increases the risk of extubation failure and subsequent re-intubation, which is associated with increased mortality and morbidity (45, 46). The optimal time window for extubation balances maximising the probability of extubation success with minimising the adverse consequences of prolonged ventilation. The rate of extubation failure increases with prematurity, rising from 20% for gestational age 28–31 weeks to >60% for < 28 weeks, but is also influenced by numerous demographic and clinical factors, and there is significant variation in practice between NICUs and individual clinicians (45, 47–51). An ability to predict extubation outcome could support clinicians to optimise the timing of extubation attempts and potentially minimise the complications of extubation failure and prolonged ventilation.

A number of predictive models have been developed to potentially aid clinicians deciding whether to attempt extubation, summarised in Supplementary Table S1 (52–67). Traditional statistical models have highlighted the value of several clinical risk factors in extubation outcome prediction and have paved the way for more recent machine learning model development (52, 53, 55, 58–66). Most existing models (both ML and conventional statistical) classify infants into binary ‘success’ and ‘failure’ groups. Importantly, the models differ in whether their modelled outcome is extubation ‘success’ or ‘failure’, and the interpretation of performance metrics such as sensitivity and specificity therefore differs from study to study. For instance, the low specificity/high false positive rate derived by Zhu et al. (where extubation *failure* is the modelled outcome) and high false negative rate in the APEX study (where *success* is the modelled outcome) both equate to infants being predicted to fail extubation who in fact had successful extubation, which could potentially result in delayed extubation and prolonged ventilation (53, 67). Conversely, the high false positive rate reported by Espindola et al. (where extubation *success* is the modelled outcome) could result in extubation attempts that are too early (54). Determining the clinical acceptability of this trade-off would require direct comparisons of the clinical impact of late vs. early extubation. Some papers therefore suggest that predictive models should not be used to solely determine extubation timing, but instead to identify infants who might require more intensive monitoring, or guide non-invasive respiratory support after extubation, for example (67).

Across studies there is also considerable heterogeneity in the exact outcome definition: there is a lack of consensus on the time window that should be used in research studies used to specify extubation outcome (68), so while some studies define ‘extubation failure’ as the need for reintubation within 72 h, others select a window of 48 h, 5 days or even 7 days (52–56, 58–67). The APEX study initially defined extubation success as the absence of specific criteria including oxygen needs, blood gases, and apnoeas post-extubation, but after noting variation in recording across different study centres, changed this to need for reintubation in 72 h, consistent with other published examples (67). Whilst some studies justify shorter reintubation time windows to avoid capturing infants who require reintubation due to a new, unforeseen clinical event, use of a shorter window may not capture the full range of infants who fail extubation. In addition, all studies assessed infants who had been selected as ‘ready’ to extubate by the clinical team, which may be subjective. Centre-specific extubation guidelines and additional factors such as caffeine or steroid administration prior to extubation, as well as post-extubation support protocols and reintubation thresholds may also impact on model interpretation and generalisability, particularly for single-centre studies.

In most of the studies referenced, combining multiple risk factors outperformed single clinical indicators. Whilst there is some overlap in the inputs used across studies (e.g., APEX (67), Espindola (54) and Gupta (58) agree on gestational age as a strong predictor, and Espindola, Gupta and Zhu (53) all select weight at extubation) there is generally considerable heterogeneity of inputs used, which can also limit comparison. The decision to extubate preterm infants is dynamic, and the contribution of risk factors varies as infants develop. Vital signs may reflect infants’ evolving physiology real-time; they are routinely collected on most NICUs and could improve the real-time accuracy of predictive models. In the multicentre APEX study, researchers collected respiratory rate, heart rate and oxygen saturations, and categorised these into respiratory patterns using an unsupervised algorithm (67). Less than 20% of the infants in this study failed extubation, leading to considerable data imbalance, which is addressed by use of a Clinical Decision-Balanced Random Forest classifier model. Using the most discriminatory features identified within this classifier along with set clinical parameters, the model predicted extubation outcome with 73% balanced accuracy (67). Interestingly, when the model was combined with clinical judgement, it improved the probability of successful extubation from 82% to 93% for the whole cohort, and from 85% to 97% for infants <7 days old, highlighting an important potential role for ML models in supporting clinician judgement, rather than as standalone decision-making tools. The reduction in accuracy after the first week of life could be related to the fact that infants ventilated for longer are often more premature and sick and therefore may have additional factors confounding their extubation outcome prediction. The fact that predictions of extubation failure within 72 h were more accurate than a longer time period in *post-hoc* analysis may limit the model's strength at detecting infants who will require delayed reintubation; however it may also reflect the most clinically unstable and therefore relevant period, since early reintubations are associated with the greatest morbidity and mortality. Moreover, the relatively high sensitivity and specificity (70% and 75% respectively) of this model could be related to the precise technologies (e.g., Respiratory Inductance Plethysmography and photoplethysmography) used, which may not be accessible in all NICUs and could restrict wider global use.

There are a large range of possible inputs available from neonatal datasets, some of which have been investigated to improve the predictive accuracy of extubation prediction models. For instance, lung and diaphragm ultrasound findings have been shown to predict extubation failure with high accuracy, with numerous studies using ultrasound scan results with conventional statistical models (predominantly logistic regression) to predict extubation outcome with apparently high accuracy (one study reported AUROC 0.98) (59–66). More recent studies have also utilised ventilator settings to improve extubation failure predictions (55, 56). Studies have shown that the ‘respiratory severity score’ (RSS), calculated from FiO2 × MAP (mean airway pressure), is associated with extubation outcome at baseline and during/after treatment with dexamethasone, suggesting a potential role for predictive modelling in guiding steroid regimens (sometimes given to intubated infants to improve extubation success) (57, 69). Gupta et al. incorporated peak RSS with clinical and laboratory parameters into their multivariable logistic regression, to develop a clinically-available “extubation readiness estimator” (58), which has been externally validated, demonstrating area under the curve of 0.77 (58, 70). This estimator's sensitivity and specificity varied considerably depending on the cut-offs for extubation readiness used (based on extubation success score), which demonstrates the importance of clearly defining how a model's output should be clinically interpreted to ensure maximum clinical benefit. Nguyen et al. additionally combined several ventilator variables with postmenstrual age to train their logistic regression model, which is also available as an online calculator tool undergoing prospective evaluation, although to the best of our knowledge, this has not been integrated into clinical practice currently (55). Brasher et al. developed multiple ML models inputting different combinations of intermittent hypoxia (derived from pulse oximetry) and SIMV (Synchronized Intermittent Mechanical Ventilation) settings (56). Model performance differed according to input parameters, and age strata used. This echoes the age-dependent model performance seen in *post-hoc* analysis of the APEX study, and highlights the importance of clearly defining the target population for such models to be clinically applied, in order to maintain reported accuracy in clinical populations (67). Notably, the majority of existing studies developing extubation prediction models were based on retrospective data collection, which can create challenges with incomplete datasets, which could impact on model accuracy. The APEX study was unique in reporting a clear strategy for handling missing data, which is listed within the PROBAST-AI framework. Although formal assessment using TRIPOD-AI and PROBAST-AI was beyond the scope of this narrative review, several of their components are reported in Supplementary Table S1.

Overall, although studies were generally well reported, many are limited by lack of external validation, prospective evaluation, or current evidence of clinical implementation, which may contribute to most remaining in the research environment currently. Nevertheless, the high performance of some models suggests the potential for predictive modelling to aid clinical decision making in this area. Going forward, consensus on a definition of extubation failure, external validation of models across different populations and inclusion of infants in studies who are not already deemed by the clinical team as ready for extubation will be important for clinical translation.

### Bronchopulmonary dysplasia

BPD is a key complication of prematurity which can result from prolonged mechanical ventilation and can impact on long-term respiratory and neurodevelopmental outcomes (72). BPD was first defined in 1967 by Northway and colleagues, who described the disease in moderately premature neonates who had received mechanical ventilation with high oxygen concentrations, and identified chest x-ray features and patterns of pathological lung injury (71). The characteristics of BPD and neonatal care have since evolved, with increasing survival of extremely preterm neonates resulting from use of antenatal steroids, neonatal practices such as surfactant used to treat RDS, and advances in respiratory support. BPD definitions have reflected the evolving phenotype and clinical practices over the last 60 years (72, 73). The National Institute of Child Health and Human Development (NICHD) Consensus definition in 2001 defined BPD as requiring oxygen supplementation for at least 28 days, and classified BPD severity into mild, moderate, or severe categories according to oxygen concentration and need for positive pressure support at 36 weeks postmenstrual age (74). This definition presented a relatively standardised international approach and has been widely adopted but has limitations due to oxygen use varying between centres, and does not accommodate newer respiratory support practices. Revised NICHD definitions from 2016 to 2018 incorporated broader respiratory support methods including nasal high flow cannula and CPAP (continuous positive airway pressure) (75). The Jensen/NICHD Neonatal Research Network 2019 definition grouped infants into grades (1–3) of BPD severity based on respiratory support mode only (76). There is currently no universally accepted definition for BPD, and as such there is significant variation in the definitions used in research models, which has implications for the comparability and longevity of models developed to predict BPD risk (77, 78).

Numerous potentially modifiable antenatal and postnatal demographic, clinical and physiological risk factors for BPD have been identified; the ability to synthesise these risk factors to predict BPD development could inform prognosis and enable early risk factor modification and personalised monitoring (79–81). Early risk stratification could also inform treatment choices: for instance, a 2014 meta-analysis showed that in infants with BPD risk <33%, postnatal steroids increased the chance of death or cerebral palsy, while when the risk of BPD was >60%, postnatal corticosteroids decreased the chance of both adverse outcomes (82).

Existing research investigating BPD prediction is summarised in Supplementary Table S2. A deep-learning model developed by Delgado-Eckert et al. was able to detect the development of BPD in the first month of life with 96% accuracy, using flow measures during tidal breathing (83). An ability to predict BPD even earlier, such as from birth, could guide respiratory support strategies early on and potentially alter disease trajectory. In 2011, Laughon and colleagues developed the first web-based BPD Outcome Estimator, based on the 2001 NICHD definition (44, 74). They developed separate logistic regression models outputting BPD risk and severity using individual and combined risk factors, at six postnatal ages starting from day 1 (44). The models showed increasing accuracy with increasing (postmenstrual age) PMA and the factor which contributed most to the model prediction also changed depending on postnatal age: whilst gestational age improved outcome prediction at day 1 and 3, subsequently respiratory support became more influential. Importantly, however, this model lacked predictive power following external validation, and was updated in 2022 to reflect the evolving definition and clinical picture of BPD, with the outcome now based on Jensen's 2019 BPD definition (76, 84). The resultant multinomial logistic regression model, developed with more than twice the number of infants than the first model, was used to create an online calculator of BPD risk according to postnatal day (84, 85). Similar to the original model, birth weight was the most important risk factor on day 1, and respiratory support became more influential on later days. The performance for this updated model were notably lower than the original model, which the authors attributed to the different BPD definitions used, different methodology used to calculate the performance and change in the patient population over time (data drift). The model was later externally validated in independent cohorts, and maintained predictive accuracy in the first of these (86). In a second cohort, use of the calculator resulted in an overestimation of BPD burden (positive predictive value 57.8%), which potentially could have been influenced by differences in the sample population (e.g., higher rate of CPAP use, different sample group demographics and differences in steroid practices) (87). The clinical implications of false positives in this context depends on the result interpretation by clinicians, but could result in over-treatment or over-investigation of infants thought to be at high risk of BPD development (89).

As understanding of BPD pathophysiology has evolved, researchers have considered further possibilities for predictive model inputs. Verder et al. included data from gastric aspirate samples combined with clinical data at birth when developing a point-of-care test based on a support vector (ML) model, predicting BPD development with 88% sensitivity and 91% specificity (17). Verder and colleagues noted that some rare heritable causes (e.g., TNF-238 polymorphisms) which increase risk of BPD were not reflected in their study population (88). This is the case for most of the studies cited here, and could result in their models not being generalisable to populations with a higher prevalence of these heritable, albeit rare, causes. More recently, Dai et al. sought to investigate the impact of selected risk genes on BPD risk prediction, and included ‘genetic risk’ identified through exome sequencing and development of a scoring system to a broad array of antenatal, perinatal and postnatal clinical features (89, 90). Addition of risk genes improved the AUROC of their predictive model from 0.814 to 0.915 and 0.826 to 0.907 for BPD and severe BPD respectively. Radio-imaging, including x-ray and ultrasound, have also been found to improve BPD diagnostic accuracy, and several ML models have incorporated chest x-ray findings, yielding predictive AUROC of up to 0.97 in one study (91–93). Ozcelik et al., for example, trained a deep-learning model using weekly recorded chest radiographs, enabling the model to learn changes and detect early BPD development (91).

Continuous vital signs parameters could similarly enable ML models to track evolving physiology and detect early signs of developing BPD with a potentially even higher resolution. Fairchild et al. found that gestational age and birth weight were highly predictive for BPD development, consistent with previously mentioned studies, but additionally incorporated number of days on mechanical ventilation and respiratory acuity score into their logistic regression model, which improved AUROC from 0.829 to 0.899 compared to using gestational age alone (94). Choi et al. hypothesised that dynamic postnatal factors, including respiratory support, FiO_2_ and blood gas results, would enhance performance of their integrated prediction model, and found that incorporating dynamic factors compared to static perinatal factors alone improved the AUROC from 0.805 to 0.912 in internal validation, which was maintained in external validation (95). Sullivan et al. additionally found that adding heart rate and oxygen saturation to clinical risk factors to produce a ‘pulse oximetry predictive score’ improved the predictive performance of their multivariate logistic regression model from AUROC of 0.921 to 0.935 in a cross-centre population of VLBW infants (96). Sullivan's study was cross-validated across two NICUs and externally validated, and is unique amongst the included studies in referencing adhering to the TRIPOD framework. It is important to note that physiological thresholds (e.g., desaturation, bradycardia and apnoea limits) often differ between studies and may therefore affect model performance and comparability.

Considerable heterogeneity in BPD definitions, prediction time points and use of severity stratifications limit the generalisability and comparability of existing models. When developing predictive models based on a clinical definition of a disease, multiple clinical phenotypes may end up being categorised within the same output group, so individual classifications should be treated with caution. For instance, using Jensen's respiratory support-based definition, respiratory support mode may be strongly influenced by local guidelines, clinician preference and equipment availability, making infant grouping centre-specific. As such, external validation is especially important for generalisability of BPD prediction models, although very few existing models have been validated in independent datasets.

A particular challenge for BPD prediction models is the consideration of death as a competing outcome. Since many infants who are at highest risk of BPD development also have highest mortality risk, excluding infants who die before 36 weeks PMA (when BPD is often diagnosed) could risk missing out infants would have developed BPD, and cause bias and underestimation of pathology. Some authors therefore include death as an outcome, such as Laughon et al., who only excluded infants who had died at <12 h of life (44). Other authors choose to assume such infants would have developed BPD, such as Fairchild et al., who included 10 babies in analysis who died after 7 days in their BPD group automatically (94). Alternatively, others do not report death as an outcome or automatically exclude infants who die; this inconsistency limits model comparability.

The majority of the cited models were developed using retrospective datasets, which can create challenges for data completeness, since BPD development and follow-up usually occur over long timeframes. The studies vary in how they report handling of missing data: Greenberg et al. excluded babies with missing data that meant BPD status could not be determined, whereas Dai et al. manually imputed missing data, which could have resulted in some inconsistencies between model performances (84, 90). Although retrospective studies provide useful information and proof-of-concept regarding the ability of ML models to predict BPD, future prospective studies in the clinical setting will be essential to determine real-time clinical utility.

### Apnoea of prematurity (AOP)

AOP affects more than 50% of preterm infants (97). It is characterised by the presence of apnoeas, commonly defined as pauses in breathing lasting at least 20 s, or at least 10 s with associated bradycardia/hypoxaemia (98, 99). Apnoea can be classified into central apnoeas (due to a lack of respiratory effort), obstructive apnoeas (airflow is blocked, despite continued respiratory effort), and mixed (components of both central and obstructive apnoeas) (100, 101). Apnoeas can be associated with acute complications including cyanosis and intermittent hypoxia, and chronic effects including poorer long-term neurodevelopmental outcomes (102, 103). AI applications have been developed to address several challenges faced with AOP: the detection of true apnoeic events, predictions of imminent apnoeas, prediction of cardiorespiratory instability as a result of apnoeas, and tailoring treatment decisions based on each infant's physiology. These are explored further below and existing models are summarised in Supplementary Table S3.

#### Detection of true apnoeas

Accurate detection of apnoea can enable treatment optimisation and improve short- and long-term outcomes (102, 103). Many preterm infants have continuous monitoring of vital signs, which often detect apnoeas through impedance pneumography, a method which utilises ECG electrodes to measure breathing activity (104, 105). A key limitation of impedance pneumography derives from the fact that it detects chest wall movement to assess apnoeas. In central apnoeas, the lack of respiratory drive leads to an absence in chest wall movement, thus leading to detection via impedance pneumography. In obstructive apnoeas however, respiratory drive, and thus chest wall movement, can persist. Most of the machine-learning papers mentioned in this section that use impedance pneumography to assess apnoeas therefore typically only assess central and mixed apnoeas (106, 107).

Built-in apnoea detection algorithms are often not validated for infants and frequently miss events, leading to inaccuracies in infants’ true and detected apnoea frequency (105, 108). In addition, built-in apnoea detection systems can have a high false-positive rate, leading to phenomena such as ‘alarm fatigue’ (109, 110). Several studies have developed ML models to identify periods of true apnoea compared with false apnoea detection (e.g. due to a period of shallow breathing, or loss of contact of the sensors) (105, 111). One of these algorithms identified true apnoeas with an accuracy of 93% and found that over 80% of the apnoeas detected by the algorithm had been missed in clinical notes (105, 112). The incorporation of such algorithms into existing bedside monitoring on NICUs could lead to fewer false positive and greater true positive apnoeic alarms, aiding clinicians to promptly identify and respond to true apnoeas. Krupa et al. sought to overcome issues with monitoring from standard neonatal sensors being used to train models (e.g., artefacts, skin damage) by developing a synthetic signal generation framework using deep learning to replicate infant respiratory cycles, suggesting that synthetic data could help bolster available real-world data for ML model development (113)*.*

#### Prediction of imminent apnoea

Predictive models have also been developed to determine infants’ unique apnoea risk, which differs based on demographic, clinical and/or physiological factors (111, 114–117). One of the first published attempts using ML models to predict apnoeic risk came from Williamson et al. in 2011 (116). They used vital signs recordings from 6 preterm infants, including inter-breath intervals (IBIs), heart rate and oxygen saturations to train ML models for each infant to produce a continuous risk score, with a high score indicating that an infant was likely approaching an apnoeic period. The average AUROC across the models was 0.73. However, only 3 of the 6 infants produced statistically significant results. A subsequent study by the same group in 2013 utilised the same population of 6 infants with the same raw cardiorespiratory data, but this time added an additional movement input from pulse plethysmograms (117). This study used a more robust ML framework, yielding a combined AUROC of 0.80, and statistically significant predictions in 5 of the 6 infants (117). The same group several years later developed a hybrid ML model (consisting of Gaussian mixture model and logistic regression) to predict apnoeic events, and found that including movement features improved the models’ predictive accuracy significantly compared to using cardiorespiratory parameters alone (118). This may be related to movement distorting respiratory signal measurement (including movement as a variable accounts for this) but also could reflect the role of movement in the pathophysiology of apnoea itself (119).

Combined models have shown promise in further studies predicting apnoeic events over longer periods of time, for example that by Xu et al., who combined LASSO (Least Absolute Shrinkage and Selection Operator) and multivariate logistic regression analyses to establish a model predicting overall apnoea risk at the time of birth/admission (120). Shirwaikar et al. took a similar approach to predict individual infants’ risk of developing apnoea using baseline parameters at birth or on admission to NICU, but using a different combination of ML models, and found that a random forest classifier showed highest performance (114). The precise clinical application of these models depends on their output: those predicting imminent apnoeas seconds before from continuous physiological signals, such as that by Zuzarte et al., could help identify the physiological transition immediately before apnoea and potentially trigger clinical review (118); longer-term apnoea risk prediction models could enable at-risk infants to be identified early, which could enable personalised monitoring, treatment and risk factor modification (114, 120).

#### Prediction of apnoea-related physiological instability

Whilst some apnoeas may be tolerated well, others are associated with desaturations, bradycardia, and in extreme cases, collapse. Although cardiorespiratory instability is associated with the duration of the apnoea, there is wide variability in the response (121). Cardiorespiratory instability following apnoea is associated with adverse neurodevelopmental outcomes, so identifying infants who are vulnerable to apnoea-related instability could help mitigate long-term effects. Chen et al. identified several clinical, demographic and physiological factors impacting apnoea-related cardiorespiratory instability (122). They used these to train a model which predicted infants’ risk of apnoea-related instability with balanced test accuracy of 66% when using clinical/demographic features only; this increased to 75.8% when physiological factors (including baseline heart rate and oxygen saturation) were included (122). AI tools predicting infants’ risk of apnoea and apnoea-related instability could enable bespoke monitoring such as personalised apnoea alarm limits or trigger earlier clinical review for at-risk infants. If a positive output (defined as a higher risk of apnoea or apnoea-related instability) triggers clinician review, then false negatives in this context could be considered more detrimental than false positives, which differs from other predictive models discussed, such as those predicting extubation outcome. For this reason, the advised clinical action resulting from a model output should be carefully determined and defined in clinical guidelines for safe integration into clinical practice.

#### Personalising treatment for apnoea of prematurity

Finally, identifying overall individualised apnoea risk could enable personalised pharmacological treatment. Caffeine, a methylxanthine which acts as a respiratory stimulant, is the cornerstone in pharmacological treatment and prevention of apnoea. Its initiation, dosing and cessation is generally based on age or weight and often dictated by local guidelines, but a more personalised approach could improve outcomes (123). Usman et al. recently used brain activity recorded during electroencephalography (EEG) to calculate functional brain age using a deep learning model, and showed that apnoea rate is more closely associated with brain age gap (the difference between functional brain age and PMA) than PMA in moderate/late preterms (124). Moreover, in an exploratory pilot study they found that infants with immature brain age had a higher rate of apnoeas and desaturations after stopping caffeine treatment compared with those with a mature brain age, suggesting that brain age calculated using ML models may be a useful biomarker for caffeine discontinuation (124).

## Discussion

This narrative review has presented an overview of some of the existing research where AI has been used to develop predictive models in neonatology, focusing on respiratory pathologies. These studies demonstrate the promise of AI tools in neonatal care and the already considerable development and progress of existing models. Most of these models demonstrate reasonable predictive performance in the research environment, but have not yet bridged the gap between research potential and clinical implementation. In this section, we will explore some key barriers to the clinical translation of neonatal predictive models and suggest steps necessary to overcome these in the future. Figure 2 summarises some steps which will be key to successfully bridging the gap between theoretical potential and real-life clinical utility for AI tools in neonatology.

**Figure 2:**
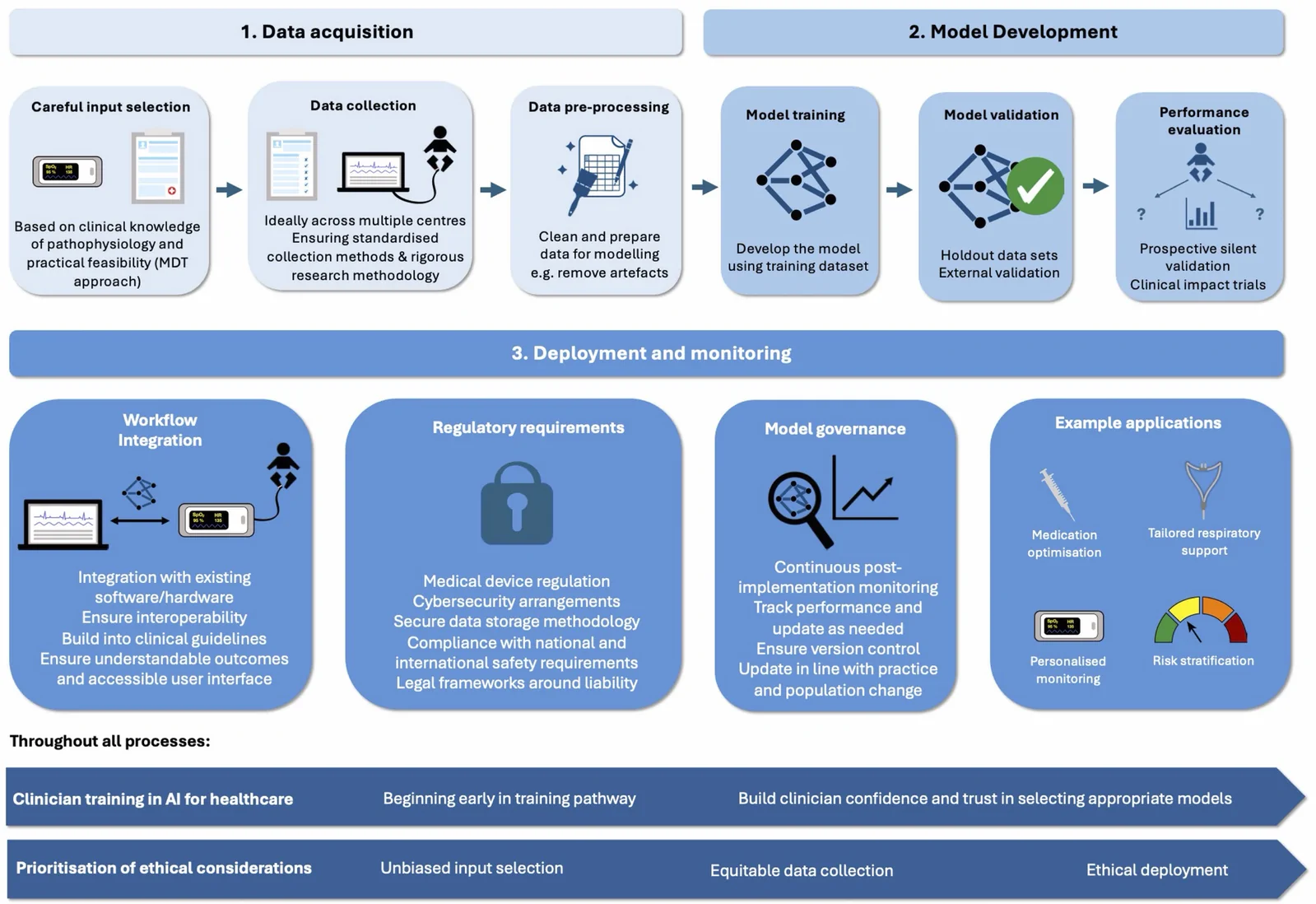
Suggested pipeline for integrating artificial intelligence (AI) tools successfully into MDT. MDT = Multidisciplinary team.

### Generalisability

Developing high-quality and generalisable ML models is challenging and many of the technical challenges summarised in Figure 1 can be exaggerated for neonatal datasets. Neonatal practice is often protocol-driven and centre-specific, and subject to individual clinician judgement and experience, so a model trained on a single-centre dataset may lack generalisability. For example, BPD prediction models which use a respiratory support mode-based definition of BPD may have centre-specific classification of participants, since mode of respiratory support is strongly influenced by local protocols, clinician experience and equipment availability (125). External validation in an independent dataset is therefore vital to ensure that model performance persists (126). Even well-validated models may still systematically over or under-estimate risk when applied to different clinical environments, due to precise combinations of patient demographics, disease prevalence, equipment or clinical practice. Prospective silent validation involves deploying a predictive model real-time within a clinical environment without displaying its output to clinicians or influencing management, to allow model predictions to be compared to clinical outcomes under real clinical conditions (126, 127). This can provide evidence that the model will perform reliably outside of the development dataset and can provide a bridge between retrospective validation and clinical implementation, while avoiding risks to patient care, and should be considered when aiming to integrate new AI tools into neonatal care. Furthermore, local calibration adjusts a predictive model to ensure that its predicted risks adequately reflect outcomes within the population it will be used in and may be a valuable tool to improve the clinical reliability of predicted probabilities (39, 128). This is especially pertinent if a model aims to be implemented internationally, as differences in neonatal/obstetric care, health systems and population demographics can result in significantly different clinical realities between populations. For instance, in high-income countries, 50% of neonatal sepsis cases are caused by Group B Streptococcus, whereas in lower-income countries, it accounts for <1% of cases, so a sepsis-prediction model aiming to be applied internationally would need to consider this (129, 130). National and international multi-centre collaboration and data sharing will be key to developing generalisable models by generating representative training and test datasets (10, 31, 131). Moreover, care needs to be taken regarding data harmonisation; for example, studies have shown significant variability in vital signs recordings and apnoea detection for infants of matched demographics, suggesting monitor variability and differences in collection methodology (108, 132, 133).

Ethical considerations are central to the development and responsible deployment of neonatal AI tools. In neonatal research, there is consistent under-representation of minority ethnicity infants, who are simultaneously more vulnerable to morbidity and overall mortality (134, 135). Active steps to avoid implicit model bias include purposive sampling of training datasets, careful handling of covariates (e.g. gestational age), and involvement of patient and public involvement (PPI) throughout model development (136, 137).

### Translation into the clinical environment

Once a predictive tool has been developed and validated to a high standard, it must be embedded into existing clinical processes, known as workflow integration. NICUs are high-pressured environments requiring rapid decision-making in a vulnerable patient population; therefore, AI tools must complement rather than complicate clinicians’ workflow. Poor clinical usability may reduce acceptance even for highly accurate models, and factors such as user interface design, cognitive workload, interpretability, training requirements, and clinician confidence can all influence successful adoption. Many excellent models are not understandable or accessible to clinicians, many do not have clear outcomes, and if they do, it is often not clear how these relate to actual treatment decisions. To be clinically implemented, model output should be easy to interpret and clinical guidelines that clearly define the AI tool's role and how the outputs should be interpreted are key (138, 139). When a defined outcome is produced, this should be displayed through an interface that is accessible and time-efficient for clinicians to use (54). Model transparency will also enable clinicians to clearly explain the use of an AI tool to parents, potentially also increasing parental confidence (139). Human factors evaluations examine specifically how clinicians interact with AI systems and whether outputs are understandable, trustworthy and usable in clinical practice (140). AI tools should not aim to supersede the role of clinician assessment, but rather to support clinicians in exercising their own judgement, and behavioural studies in parallel with model development can ensure AI tools are aligned with pre-existing clinician reasoning patterns (54, 141). Moreover, clinical impact trials, which evaluate whether implementing an AI tool improves patient outcomes, are key to determine benefits (or risks) of use and should be considered a pre-requisite for the widespread adoption of AI tools in neonatal care (10). Compared to validation studies, which assess model accuracy, clinical impact trials assess whether the predictive tool changes clinical practice and benefits patients, and can be a key step in ensuring clinician confidence and uptake. AI tools can feel overwhelming and inaccessible for clinicians who lack exposure, and incorporating dedicated training on AI early in clinical training may empower clinicians (142).

As well as integrating within a single centre's workflow, for use across different organisations, a model should demonstrate interoperability, which describes the ability of AI tools to interact with clinical equipment (e.g. bedside monitors, ventilators), laboratory result systems, electronic record systems and other medical devices across different healthcare systems (143). Since many neonatal ML models rely on assimilation of multimodal demographic, clinical and physiological parameters, interoperability can be a particular challenge, and can prevent successful across-centre implementation. Model development using multicentre datasets, and adoption of standardised healthcare data formats is key to facilitating widespread integration (144, 145). If the model involves use of a new medical device, then this must comply with national or international regulatory requirements before integration into clinical use (146). Regulatory agencies require evidence that models are safe, clinically effective, reliable and appropriately managed throughout their lifecycle, and compliance with such systems is a vital component of ensuring that future neonatal AI systems meet recognised standards for safety, quality, and clinical performance (144–146). In addition to the standard regulatory requirements for medical devices, AI tools in neonatal care may involve the assimilation of large quantities of sensitive patient data, and so require close regulation in terms of data protection and cybersecurity. Robust cybersecurity safeguards and methods to maintain privacy such as de-identification and secure data storage will be necessary to enable collaborative model development whilst reducing data protection risks, and maintaining clinician and public confidence (147). AI systems integrated with hospital information systems may introduce additional vulnerabilities if not adequately secured. Regulatory authorities increasingly recognise that AI tools require thorough and repeated evaluation beyond traditional medical devices, especially since models may change and evolve over time (146).

### Maintaining model performance

Neonatal medicine is a dynamic and rapidly evolving field. Model drift describes the gradual deterioration in model performance, which neonatal AI models are particularly at risk of, due to a quickly evolving patient population, clinical practices, monitoring technologies, and research advances (144, 146). Detecting model drift requires continuous evaluation of model performance and predefined processes for recalibration and retraining (152). Continuous post-implementation monitoring is increasingly recognised as an essential component of safe and sustainable AI deployment in a clinical environment, and can include repeated evaluations of predictive accuracy, fairness across patient subgroups, technical reliability, clinician utilisation and unintended consequences of model use (e.g. alarm fatigue) (153). When unintended consequences do occur as a result of model use, it is vital that liability is clearly outlined, and there is debate regarding whether responsibility should lie with clinicians, healthcare organisations, software developers or device manufacturers when AI recommendations are followed or ignored and patient harm results (39). For neonatal AI models to become more universally integrated into routine care, clear legal frameworks around liability will be necessary, for example in situations where model output clashes with clinician judgement (145, 148). Since AI is currently intended to support rather than replace clinician judgement, clinicians generally retain ultimate responsibility for patient management, but this can be a point of concern for clinicians if they do not fully understand or trust the model they are using, and may limit clinical uptake. Overall, careful consideration of model governance will be essential to ensure that AI models are safely integrated into the care of a vulnerable patient population, and maintain the trust and confidence of the clinicians using them (145). The FUTURE-AI guideline was established by an international consortium based on 6 guiding principles—fairness, universality, traceability, usability, robustness, and explainability—and includes recommendations over the entire life cycle of healthcare AI models, including design, development, and validation, deployment, and monitoring (144). Additionally, in 2025, the International Medical Device Regulators Forum (IMDRF) released a guidance document identifying 10 guiding principles to inform the development of ML models for medical devices (146). Adherence to international consensus guidelines will help to regulate and standardise the integration of AI tools in neonatal care.

### The HeRO monitor: a key example of algorithmic predictive monitoring in neonatal clinical practice

The HeRO monitor is an excellent, but rare, example of an algorithmic predictive monitoring tool that has been successfully integrated into neonatal clinical practice, following external validation, and rigorous testing in large randomised control trials (149). Importantly, the HeRO algorithm was one of the earliest examples of continuous predictive analytics in neonatology and yet overcame many of the challenges described in this review (149–154). Exploring how this was made possible provides a framework for future clinical decision support tools. The HeRO algorithm sought to address challenges in neonatal sepsis diagnosis, a prominent cause of morbidity and mortality particularly amongst preterm infants. Early detection and subsequent treatment of neonatal sepsis is challenging; clinical signs and laboratory testing can often be unreliable, resulting in frequent late detection and thus increased mortality, but simultaneously in over-investigation and treatment of clinically-well infants (155–157). The HeRO algorithm incorporates a heart rate characteristics index derived from continuous vital signs to calculate risk of deterioration and mortality due to sepsis (150, 153, 158–162); as such it uses a widely-available and non-invasive input and has a clear, defined outcome which is closely tied to a clinical question (sepsis prediction). Using well-established physiological principles to determine inputs potentially increases clinician confidence in its reliability and clinical relevance. The HeRO algorithm fits directly into existing patient monitoring and complements existing clinical workflows, therefore reducing requirements for manual data collection and associated discrepancies (149–154). The algorithm was developed and externally validated across real-world populations, ensuring its generalisability (153). Additionally, the HeRO trial, a large-scale randomised controlled trial, included infants from 9 NICUs across the US, reducing the effect of centre and clinician-specific practices and robustly demonstrating that algorithm accuracy was maintained across different units (163). The HeRO trial showed a statistically significant reduction in all-cause mortality in VLBW infants whose HeRO score was displayed to clinicians, in comparison to those whose scores were not. It is worth noting however, that the trial's primary outcome, which looked at days alive and ventilator-free, did not yield a statistically significant result (163). Though the trial measured all-cause mortality rather than sepsis-related mortality specifically, a plausible interpretation of this is that the reduction seen was due to earlier diagnosis and treatment of sepsis. The HeRO score does not categorise patients itself, rather it produces an index score that continually updates based on the infant's heart rate variability, and still relies on clinicians to make educated decisions regarding investigating and treating sepsis. As such, it is a key example of how predictive tools, including AI-assisted clinical decision support tools, can play a valuable role in supporting, rather than superseding, clinician decision-making; this factor may also have been influential in increasing clinician trust and enabling clinical translation.

## Conclusion

In summary, the integration of AI tools into routine neonatal care could represent an opportunity to transform the care of preterm infants. Existing research demonstrates the potential of ML models for the prediction of extubation readiness, BPD, and apnoea detection and risk. Whilst numerous barriers have so far limited the widespread implementation of AI tools into routine neonatal care, there is a potential roadmap to successfully bridging the gap from research potential to clinical translation. By rapidly assimilating complex, multi-dimensional data, integration of AI tools into neonatal care in the future could support personalised monitoring and treatment to aid clinicians caring for our most vulnerable infants.
