# Supplementary material for: How could artificial intelligence aid clinicians caring for preterm neonates with respiratory pathology?
Source: Front Pediatr. 2026 Oct 13;14:1885589. doi: 10.3389/fped.2026.1885589 (PMC13711111; doi:10.3389/fped.2026.1885589)
Supplement: Supplementary file 1 [file Table1.docx]

**Supplementary Material: How could artificial intelligence aid clinicians caring for preterm neonates with respiratory pathology?**

Vithushanan Ketheeswaranathan, Surina Fordington, Caroline Hartley

**Supplementary Table 1:** **Summary of the key characteristics of the referenced models predicting extubation outcome.**

Papers are listed in date order of publication. Blue shading indicates a conventional statistical model, green shading indicates a machine learning model.
Input Timeframe = Time window over which input variables were collected or derived before the model generates a prediction
Prediction Horizon = The interval between the time at which the model made a prediction and the time at which the predicted outcome was expected to occur (also referred to as ‘Forecast Horizon)
GA = Gestational age, BW = birth weight, LUS = lung ultrasound, PDA = patent ductus arteriosus, FiO2 = fraction of inspired oxygen, PPV = positive predictive value, NPV = negative predictive value), (N)RDS = (newborn) respiratory distress syndrome, MV = mechanical ventilation, SIMV = Synchronized Intermittent Mechanical Ventilation, IVH = intraventricular haemorrhage

| **First author (year of publication)** | **Study population** | **Exclusion criteria** | **Sample size** | **Input variables** | **Data collection methodology** | **Model type** | **Outcome definition** | **Input timeframe** | **Prediction horizon** | **Validation method** | **Performance** | **Implementation status** |
| --- | --- | --- | --- | --- | --- | --- | --- | --- | --- | --- | --- | --- |
| **Gupta et al. (2019) (58)** | Preterm infants with BW <1250g, received invasive MV, first extubation attempt <60 days of life | Death before extubation, missing data regarding extubation outcome, already part of multicentre extubation readiness study | 312 infants  (single centre) | Peak RSS, gestational age, chronological age, weight, blood pH | Retrospective | Conventional statistical prediction model  (Multivariate logistic regression) | Modelled outcome = extubation success  Extubation failure > requiring reintubation within 5 days of extubation | Prior to extubation attempt | 5 days after extubation | Internal validation (bootstrap resampling) | 60% cut-off:  Sensitivity 87%*  Specificity 53%  70% cut-off:  Sensitivity 76%*  Specificity 66%  80% cut-off:  Sensitivity 54%*  Specificity 81%  AUROC 0.77  * proportion correctly identified as extubation *success* | Clinically available (online calculator) |
| **Liang et al. (2021) (59)** | GA <37 weeks, RDS diagnosed, MV for >24 hours | Congenital heart disease, respiratory malformation, and chromosome abnormality, pneumothorax, air leak, or meconium aspiration, arrhythmias and haemodynamic instability, >stage III IVH, taking other experimental drugs or participating in other clinical trials, respiratory distress from other causes, incomplete data | 314 infants (single centre) | Combined model: GA, body length, BW and LUS score  LUS score alone | Retrospective | Conventional statistical prediction model  (multivariate logistic regression) | Modelled outcome = extubation failure  Extubation failure> requiring reintubation within 48 hours of extubation | Prior to extubation | 48 hours after extubation | Internal validation reported | Combined model:  Sensitivity 86.67%*  Specificity 74.31%  AUROC 0.871  LUS only:  Sensitivity 84%  Specificity 80.69%  AUROC 0.858  *proportion correctly identified as extubation *failure* | Research |
| **Mohsen et al. (2022) (60)** | Preterm infants <28 weeks, receiving MV for >12 hours | Congenital or chromosomal abnormalities, pneumothorax, pleural effusion, intubated for non respiratory causes, receiving muscle relaxants | 45 infants (single centre) | LUS score | Prospective | Conventional statistical prediction model  (binary logistic regression) | Modelled outcome = extubation failure  Extubation failure> requiring reintubation within 72 hours of extubation | Ultrasound performed 1 hour before extubation | 72 hours after extubation | No internal or external validation reported | Lung ultrasound score ≥15 alone:  Sensitivity 95%*  Specificity 85%  AUROC 0.95  *proportion correctly identified as extubation *failure* | Research |
| **Kanbar et al. (2023) [APEX study] (67)** | Preterm infants with BW <1250g, receiving MV, first extubation attempt | Unplanned extubation, major congenital anomalies, major heart defects, cardiac arrhythmia, use of vasopressor or sedative drugs at extubation, extubated from HFOV or directly to oxyhood or low-flow nasal cannula | 241 infants  (multicentre 5 units) | Respiratory rate, heart rate, oxygen saturations (unsupervised patterns) and clinical parameters | Prospective | Supervised machine learning (Decision Balanced Random Forest Classifier) | Modelled outcome = extubation success  Extubation failure > requiring reintubation within 72 hours of extubation | Prior to extubation attempt | 72 hours after extubation | Cross-validation | Sensitivity 70%*  Specificity 75%  False positives 25%  False negatives 30%  Balanced accuracy 73%  * proportion correctly identified as extubation *success* | Research |
| **Brasher et al. (2024) (56)** | Preterm infants <30 weeks GA | Not specified | 110 infants  (single centre) | Model 1: Intermittent hypoxia + SIMV  Model 2:  Intermittent hypoxia  Model 3:  SIMV | Prospective | Supervised machine learning (Random Forest, XGBoost, SGDClassifier, Bagging) | Modelled outcome = extubation failure  Extubation failure > requiring reintubation within 7 days of extubation | At time of extubation | 7 days after extubation | 3-fold cross-validation | **Model 1 (IH + SIMV):**  All ages > Random Forest:  Sensitivity 59%, Specificity 91%, AUROC 0.77  <2 weeks > Random Forest:  Sensitivity 78%, Specificity 85%, AUROC 0.94  >2 weeks > XGBoost:  Sensitivity 42%, Specificity 97%, AUROC 0.83  **Model 2 (IH):**  All ages > SGDClassifier:  Sensitivity 47%, Specificity 81%, AUROC 0.64  <2 weeks > XGBoost:  Sensitivity 56%, Specificity 72%, AUROC 0.77  >2 weeks > XGBoost:  Sensitivity 17%, Specificity 91%, AUROC 0.73  **Model 3 (SIMV):**  All ages > Random Forest:  Sensitivity 13%, Specificity 88%, AUROC 0.71  <2 weeks > Bagging:  Sensitivity 80%, Specificity 78%, AUROC 0.86  >2 weeks > XGBoost:  Sensitivity 36%, Specificity 92%, AUROC 0.70 | Research |
| **Nguyen et al. (2024) (55)** | Preterm infants <33 weeks GA, receiving MV >24 hours | Non‐respiratory reasons for extubation failure, birth defects affecting respiratory functions, subglottic stenosis or tracheomalacia, and acute surgery | 81 infants (single centre) | PMA, 4 ventilator variables (Cdyn/kg, %MVspon, Cdyn/kg 24 hour variability, MAwP) | Retrospective | Conventional statistical prediction model  (logistic regression) | Modelled outcome = extubation success  Extubation failure > requiring reintubation within 120 hours of extubation | Prior to extubation | 120 hours after extubation | No internal or external validation reported | 60% cut-off:  Sensitivity 80%*  Specificity 73%  70% cut-off:  Sensitivity 74% *  Specificity 78%  80% cut-off:  Sensitivity 65%*  Specificity 86%  90% cut-off:  Sensitivity 52%*  Specificity 93%  AUROC 0.889  *proportion correctly identified as extubation *success* | Research  Clinical calculator developed  (undergoing prospective evaluation, no identified published evidence of clinical use) |
| **Rojas et al. (2025) (61)** | Neonates receiving MV for at least 48 hours and considered ready for extubation | Chest deformities, complex congenital heart disease, or respiratory failure of central origin | 55 infants (single centre) | LUS score  (cGA strongest predictor for <28 weeks GA) | Prospective | Multivariate Poisson regression | Modelled outcome = extubation failure  Extubation failure> requiring reintubation (or non-invasive ventilation if >28 weeks) within 72 hours of extubation | Ultrasound performed immediately before extubation | 72 hours after extubation | No internal or external validation reported | Whole cohort:  Sensitivity 88.5%*  Specificity 37.9%  AUROC 0.61  Subgroup GA ≥ 28 weeks:  Sensitivity 87%  Specificity 100%  AUROC 0.91  Subgroup GA < 28 weeks:  (LUS) AUROC 0.38  (cGA) Sensitivity 100%  Specificity 94.7%  AUROC 0.95  *proportion correctly identified as extubation *failure* | Research |
| **Xie et al. (2025) (62)** | Neonates receiving MV and planning to be weaned from the ventilator | Severe congenital malformations, chromosomal or genetic diseases, thoracic space-occupying disease, intubation for non-respiratory reasons, missing data | 92 infants (single centre) | LUS score | Prospective | Conventional statistical prediction model  (logistic regression) | Modelled outcome = extubation failure  Extubation failure> requiring reintubation within 48 hours of extubation | Ultrasound performed before extubation | 48 hours after extubation | No internal or external validation reported | Without grouping according to GA:  Sensitivity 83.3%*  Specificity 94.6%  AUROC 0.905  *proportion correctly identified as extubation *failure* | Research |
| **Espindola et al. (2025) (54)** | Preterm infants < 37 weeks GA, receiving >24 hours of MV, first extubation attempt | Transferred from another institution, required inter-hospital/unit transfer, passed away during data collection, PTNB with anomalies, syndromes, congenital heart defects, other surgical conditions | 405 infants (multicentre 6 units) | Congenital infections (STORCH), GA, BW, extubation weight, duration of invasive mechanical ventilation | Retrospective | Supervised machine learning | Modelled outcome = extubation success  Extubation failure > requiring reintubation within 48 hours of extubation | Prior to extubation attempt | 48 hours after extubation | Internal validation using multicentre dataset | Sensitivity 79.41%*  Specificity of 60%  False positives 40%  False negatives 20.59%  Accuracy (total number of correct predictions / total number of predictions made) 77.78%  * proportion correctly identified as extubation *success* | Research  (development of a user interface in progress) |
| **Zong et al. (2025) (63)** | Preterm infants with GA ≤ 25+6 who received MV for ≥72 hours | Congenital heart disease, other congenital or chromosome abnormalities, pleural effusion, pneumothorax, death before extubation, surgical, small for GA, accidental extubation, intubated for nonrespiratory causes | 93 infants  (single centre) | LUS score | Prospective | Conventional statistical prediction model  (logistic regression) | Modelled outcome = extubation success  Extubation failure > requiring reintubation within 72 hours of extubation | Daily ultrasound scans prior to extubation | 72 hours after extubation | Internal validation (bootstrap resampling) | Sensitivity 94.6%*  Specificity 92.7%  AUROC 0.98  *proportion correctly identified as extubation *success* | Research |
| **Zhu et al. (2025) (53)** | Preterm infants </=32 weeks GA with NRDS and receiving MV | Admitted >3 days of life or lasting <7 days, death or withdrawal pre-extubation, complex congenital heart disease, major malformations, or metabolic disorders, transferred for surgical care, extubation against medical advice, incomplete clinical data | 290 infants  (single centre) | Maternal ureaplasma urealyticum infection, placental abruption, extubation weight, IVH, PDA diameter | Retrospective | Conventional statistical prediction model  (Nomogram-based) | Modelled outcome = extubation failure  Extubation failure> requiring reintubation within 72 hours of extubation | Prior to extubation attempt | 72 hours after extubation | No internal or external validation | Sensitivity 91%*  Specificity 52%  False positives 48%  False negatives 9%  PPV 0.87  NPV 0.60  AUROC 0.78  * proportion correctly identified as extubation *failure* | Research |
| **Gautam et al. (2025) (64)** | Preterm infants <34 weeks GA, >24 hours MV | Major  congenital anomalies, diaphragmatic paralysis or defects,  chest deformities, prior extubation failure, intubation for neurological or  haemodynamic aetiologies | 45 infants  (single centre) | LUS  aeration score | Prospective | Conventional statistical prediction model  (logistic regression) | Modelled outcome = extubation failure  Extubation failure> requiring reintubation within 72 hours of extubation | Ultrasound performed 1 hour prior to extubation | 72 hours after extubation | No internal or external validation reported | Sensitivity 80%*  Specificity 96%  AUROC 0.97  *proportion correctly identified as extubation *failure* | Research |
| **Ibraheem et al. (2025) (65)** | Preterm infants <30 weeks GA, within first 24 hours of life, receiving MV | Congenital or chromosomal abnormalities, grade IV IVH, pneumothorax, pleural effusion, receiving sedation or muscle relaxant | 38 infants (single centre) | LUS score (model adjusted for GA, pre-extubation weight, mean airway pressure) | Prospective | Conventional statistical prediction model  (multivariate logistic regression) | Modelled outcome = extubation failure  Extubation failure> requiring reintubation within 72 hours of extubation | Prior to extubation | 72 hours after extubation | No internal or external validation reported | LUS as independent predictor:  OR 1.27, 95% CI (1.04–1.56), p = 0.02 | Research |
| **Tan et al. (2025) (52)** | Very low BW (<1500g) infants receiving MV | Death or withdrawal of care before extubation, presence of complex congenital heart disease, severe malformations, or genetic/metabolic disorders, missing clinical data | 265 infants (single centre) | GA, BW, PDA diameter, haematocrit before extubation, sepsis | Retrospective | Conventional statistical prediction model  (nomogram-based) | Modelled outcome = extubation failure risk  Extubation failure> requiring reintubation within 72 hours of extubation | Prior to extubation attempt | 72 hours after extubation | Internal (bootstrap resampling) and external validation (2 institutions) | Sensitivity 89.8%*  Specificity 77.8%  False positives 22.2%  False negatives 10.2%  Internal AUROC 0.88  External AUROC 0.862  Concordance index 0.888 (internal validation)  Concordance index 0.962 (external validation)  *proportion correctly identified as extubation *failure* | Research |
| **De Alencar Nelo et al. (2026) (66)** | Infants <2 years of age, MV for >24 hours acceptable arterial blood gas, reduced specified ventilatory support parameter | Pleural pathologies impairing diaphragmatic assessment, Zika virus, COVID-19, rhinorrhoea, or abdominal pain, severe neurological disorders, significant haemodynamic congenital heart disease, genetic syndromes, haemodynamic instability | 38 infants  (bicentric, 2 hospitals NICU and PICUs) | LUS features:  Diaphragmatic excursion, diaphragmatic contraction velocity, diaphragmatic relaxation velocity | Prospective | Conventional statistical prediction model  (binary logistic regression) | Modelled outcome = extubation success  Extubation failure > requiring reintubation within 48 hours of extubation | Ultrasound performed 5 minutes prior to extubation | 48 hours after extubation | No internal or external validation reported | Restricted bank:  Sensitivity = 0.750*  Specificity = 0.800  AUROC = 0.743 Accuracy = 0.758  Complete bank:  Sensitivity = 0.85  Specificity = 0.600  AUROC = 0.754  Accuracy = 0.789  *proportion correctly identified as extubation *success* | Research |
