# Supplementary material for: How could artificial intelligence aid clinicians caring for preterm neonates with respiratory pathology?
Source: Front Pediatr. 2026 Oct 13;14:1885589. doi: 10.3389/fped.2026.1885589 (PMC13711111; doi:10.3389/fped.2026.1885589)
Supplement: Supplementary file 2 [file Table2.docx]

**Supplementary Material: How could artificial intelligence aid clinicians caring for preterm neonates with respiratory pathology?**

Vithushanan Ketheeswaranathan, Surina Fordington, Caroline Hartley

**Supplementary Table 2:** **Summary of the key characteristics of the referenced models predicting bronchopulmonary dysplasia.**

Papers are listed in date order of publication. Blue shading indicates a conventional statistical model, green shading indicates a machine learning model and red shading indicates a deep learning model. Input Timeframe = Time window over which input variables were collected or derived before the model generates a prediction; Prediction Horizon = The interval between the time at which the model made a prediction and the time at which the predicted outcome was expected to occur (also referred to as ‘Forecast Horizon)

GA = Gestational age, BW = birth weight, PDA = patent ductus arteriosus, FiO2 = fraction of inspired oxygen, PPV = positive predictive value, NPV = negative predictive value), (N)RDS = (newborn) respiratory distress syndrome, MV = mechanical ventilation, SIMV = Synchronized Intermittent Mechanical Ventilation, IVH = intraventricular haemorrhage, FTIR = Fourier transform infrared spectroscopy

† Only conference abstract was accessible, therefore some methodology data is limited. This was felt to still be a useful study to include in this discussion.

| **First author (year of publication)** | **Study population** | **Exclusion criteria** | **Sample size** | **Input variables** | **Data collection methodology** | **Model type** | **Outcome definition** | **Input timeframe** | **Prediction horizon** | **Validation method** | **Performance** | **Implementation status** |
| --- | --- | --- | --- | --- | --- | --- | --- | --- | --- | --- | --- | --- |
| **Laughon et al. (2011) (44)** | Preterm 23-30 weeks GA, survived >12 hours, BW 401–1,250 g | <23 weeks GA,  >30 weeks GA,  death <12 hours | 3629 infants (multicentre, 17 units) | Gestational age, birth weight, race/ethnicity, sex, respiratory support, FiO2 | Prospective | Conventional statistical prediction model  (Multinomial logistic regression) | Development of BPD (defined as none, mild, moderate and severe, according to the NICHD Consensus Definition) and death | Postnatal days 1, 3, 7, 14, 21, 28 | Until 36 weeks PMA (as per NICHD BPD definition) | Internal validation (holdout dataset) and external validation (noted lack of power) | AUROC (C-statistic):  Internal validation:  Day 1: 0.795  Day 3: 0.810  Day 7: 0.823  Day 14: 0.834  Day 21: 0.848  Day 28: 0.854  External validation:  Day 1: 0.720  Day 3: 0.732  Day 7: 0.736  Day 14: 0.761 | Clinically available online calculator |
| **Sullivan et al. (2018) (97)** | Very low BW (<1500g) infants | Cyanotic congenital heart defects, comfort care only | 778 infants (multicentre 2 units) | POPS (Pulse oximetry predictive score) =  Pulse oximetry score (HR and SpO2) + clinical risk factors (including GA, BW, APGARs, antenatal steroids, sex) | Retrospective | Conventional statistical prediction model  (Multivariate logistic regression) | BPD development | First 7 days of life | Until 36 weeks PMA | Internal, cross-validation and external validation | Clinical predictive score only:  AUROC 0.921  Pulse oximetry only:  AUROC 0.886  POPS (combined clinical + oximetry):  AUROC 0.935 | Research |
| **Fairchild et al. (2019) (95)** | VLBW infants, 23-33 weeks GA | Died within the first week, severe syndromes or congenital anomalies likely to impact oxygenation, <7 days of pulse oximetry data | 502 infants (single centre) | GA, BW, ventilator days, RAS (Respiratory Acuity Score, FiO2 x numerical level of respiratory support on two days) | Prospective | Conventional statistical prediction model  (Multivariate logistic regression) | Development of BPD (defined as need for supplemental oxygen/positive airway pressure at 36 weeks PMA) and severe BPD (defined as requiring >30% supplemental oxygen at 36 weeks PMA) | 4 weeks after birth | Until 36 weeks PMA | None reported | BPD predictions:  GA alone:  AUROC 0.829  GA+RAS28:  AUROC 0.889  GA+RAS28+%time in desaturation events <80% for ≥10 seconds:  AUROC 0.902  Severe BPD predictions:  GA alone:  AUROC 0.842  GA+RAS28:  AUROC 0.913  GA+RAS28+%time in desaturation events <80% for ≥10 seconds:  AUROC 0.923 | Research |
| **Verder et al. (2021) (17)** | Preterm infants (23-31 weeks GA) | Not specified | 61 infants (single centre) | FTIR spectral data analysis from gastric aspirates, gestational age, birth weight, surfactant treatment | Prospective | Supervised machine learning (support vector machine) | Development of BPD (defined as none, mild, moderate and severe, according to the NICHD Consensus Definition) | At birth | Until 36 weeks PMA (as per NIH BPD definition) | Cross-validation | Sensitivity 88%  Specificity 91%  AUROC | Research |
| **Dai et al. (2021) (91)** | Preterm infants (<32 weeks) | Major congenital malformations, clinical syndromes, chromosomal abnormalities, systemic infections and shock, or other definite diseases beyond respiratory, did not undergo exome sequencing, died within 7 days of birth | 245 infants (single centre) | Exome sequencing of identified ‘risk genes’, 21 clinical features measured | Prospective | Supervised machine learning model (LASSO) | Development of BPD and severe BPD (definition not specified) and death | Exome sequencing within 14 days of birth; clinical features up to 36 weeks PMA | Until 36 weeks PMA (as per NIH BPD definition) | Cross-validation | BPD model:  AUROC (clinical only) 0.814  AUROC (+risk genes) 0.915  Severe BPD model:  AUROC (clinical only) 0.826  AUROC (+risk genes) 0.907 | Research |
| **Greenberg et al. (2022) (85)** | Extremely preterm infants (GA 23–28 weeks, 501-1250g BW) | Death ≤12 hours after birth, major congenital anomalies, < 36 weeks PMA, missing data to determine BPD status, and admission to a NICU with <20 infants meeting inclusion criteria | 9181 (multicentre) | Gestational age, birth weight, race/ethnicity, sex, respiratory support, antenatal steroids, surgical NEC | Prospective  (retrospective analysis) | Conventional statistical prediction model  (Multinomial logistic regression) | Development of BPD (defined as none, grade 1, 2 or 3 according to Jensen’ et al’s 2019 respiratory support mode-based definition | Postnatal days 1, 3, 7, 14, 21, 28 | Until 36 weeks PMA (as per 2019 BPD definition) | External validation (by different groups) | AUROC (C-statistic)  Day 1: 0.674  Day 3: 0.686  Day 7: 0.692  Day 14: 0.702  Day 28: 0.741 | Clinically available (online calculator) |
| **Xing et al. (2022) (93)** | Premature infants (GA not specified) | Not specified | 121 infants (single centre) | CXR findings | Prospective | Deep learning model (XSEG-NET) | Development of BPD (defined as mild, moderate and severe, according to the NICHD Consensus Definition) | Day 28 of life | Until 56 days of life | Cross-validation | Sensitivity 95.67%  Specificity 96.98%  Accuracy 95.58%  Precision 95.61% | Research |
| **Delgado-Eckert et al. (2024) (84)** | Preterm infants^†^ | Severe maternal health problems, major birth defects or perinatal disease e.g. respiratory distress, airway malformation, major respiratory disease | 190 infants (multi- centre) | Time series measurements during tidal breathing^†^ | Prospective | Deep learning (artificial neural network) | BPD development and severity^†^ | 44.7 weeks^†^ PMA (average) | Not specified^†^ | Internal validation (holdout dataset) | Sensitivity 96%  Specificity 100%  Accuracy 96% | Research |
| **Choi et al. (2025) (96)** | Preterm infants (<32 weeks GA) | Congenital heart defects, severe chromosomal abnormalities, or major malformations that could affect baseline saturation levels | 621 infants (single centre) | Static clinical and demographic factors + VentFiO2 + blood gas analysis | Retrospective | Supervised machine learning (Random Forest Classifier) | Development of moderate or severe BPD (defined according to the 2001 NICHD Consensus Definition) | First week of life | Until 36 weeks PMA | Cross-validation, internal and external validation | Combined model (best):  AUROC 0.841  Internal validation:  AUROC 0.912  External validation:  AUROC 0.814 | Research |
| **Ozcelik et al. (2025) (92)** | Preterm infants ≤ 28 weeks GA and < 1250 g BW | Major congenital abnormalities, did not survive the first 28 days | 122 infants (single centre) | CXR findings (labelled as BPD-0 no BPD, BPD-1 mild BPD, BPD-2 moderate BPD, and BPD-3 severe BPD) | Retrospective | Deep learning | Development of BPD, classified as severe, moderate, mild and no BPD (according to 2018 Eunice Kennedy Shriver National Institute of Child Health and Human Development criteria) | Week 1, week 2, week 3 and week 4 of life | Until 36 weeks PMA (as per NICHD BPD definition) | Internal validation (holdout dataset), | Week 1:  No BPD: AUROC 0.82  Sensitivity 51.5% Specificity 92.1%  Mild BPD: AUROC 0.82  Sensitivity 79.2 %Specificity 76.5%  Moderate BPD: AUROC 0.75  Sensitivity 28.6% Specificity 95.7%  Severe BPD :AUROC 0.79  Sensitivity 73% Specificity 80%  Week 4:  No BPD AUROC 0.97  Sensitivity 50% Specificity 95.7%  Mild BPD AUROC 0.86  Sensitivity 53.8% Specificity 91.9%  Moderate BPD AUROC 0.82  Sensitivity 52.4% Specificity 98.1%  Severe BPD AUROC 0.90  Sensitivity 94.3% Specificity 70% | Research |
