# Supplementary material for: How could artificial intelligence aid clinicians caring for preterm neonates with respiratory pathology?
Source: Front Pediatr. 2026 Oct 13;14:1885589. doi: 10.3389/fped.2026.1885589 (PMC13711111; doi:10.3389/fped.2026.1885589)
Supplement: Supplementary file 3 [file Table3.docx]

**Supplementary Material: How could artificial intelligence aid clinicians caring for preterm neonates with respiratory pathology?**

Vithushanan Ketheeswaranathan, Surina Fordington, Caroline Hartley

**Supplementary Table 3:** **Summary of the key characteristics of the referenced models relating to apnoea.**

Papers are listed in date order of publication. Blue shading indicates a conventional statistical model, green shading indicates a machine learning model.
Input Timeframe = Time window over which input variables were collected or derived before the model generates a prediction; Prediction Horizon = The interval between the time at which the model made a prediction and the time at which the predicted outcome was expected to occur (also referred to as ‘Forecast Horizon)

GA = Gestational age, BW = birth weight, PDA = patent ductus arteriosus, FiO2 = fraction of inspired oxygen, IBI = interbreath interval, PPV = positive predictive value, NPV = negative predictive value, (N)RDS = (newborn) respiratory distress syndrome, MV = mechanical ventilation, SIMV = Synchronized Intermittent Mechanical Ventilation, IVH = intraventricular haemorrhage.

| **First author (year of publication)** | **Clinical aim** | **Study population** | **Exclusion criteria** | **Sample size** | **Input variables** | **Data collection methodology** | **Model type** | **Outcome definition** | **Input timeframe** | **Prediction horizon** | **Validation method** | **Performance** | **Implementation status** |
| --- | --- | --- | --- | --- | --- | --- | --- | --- | --- | --- | --- | --- | --- |
| **Adjei et al. (2021) (106)** | Apnoea detection | Preterm infants (part of 2 separate studies involving vital signs recording, and ROP data collection study) | Not specified | 42 infants (single centre) | Impedance pneumography data (+/- oxygen saturation + heart rate) | Retrospective | Machine learning model (support vector machine) | Interbreath interval (apnoea detection) | Continuous recording | Not applicable  (not forecasting a future event) | Cross-validation and external validation | IP signal lone:  Accuracy 75%  Specificity 62%  IP signal + HR + sats:  Accuracy 87%  False positives 5%  False negatives 16%  External validation:  Accuracy 93%  False positives 5%  False negatives 16% | Research |
| **Varisco et al. (2022) (112)** | Apnoea detection | Preterm infants <30 weeks GA, who developed late onset sepsis and case-matched controls | Not specified | 20 infants (single centre) | IBIs, signal instability index (used to extract 47 features) | Retrospective | Conventional statistical prediction (logistic regression) and machine learning (random forest and support vector machine) | Central apnoea detection (vs false alarm) | Continuous recording (features extracted from 4 overlapping 30 second time windows) | Not applicable  (not forecasting a future event) | Cross-validation (LOPO-CV and 10-fold) | Logistic regression (highest accuracy):  LOPO-CV (leave one patient out cross-validation): AUROC 0.88  10-fold cross-validation: 0.90  Support vector model:  LOPO-CV: AUROC 0.87  10-fold cross-validation: 0.85  Random Forest:  LOPO-CV: AUROC 0.89  10-fold cross-validation: 0.87 | Research |
| **Williamson et al. (2011) (117)** | Apnoea prediction | Preterm infants (25.4-31 weeks GA, 0.78-1.5kg BW) | Not specified | 6 infants  (single centre) | IBIs, heartbeat intervals | Retrospective | Machine learning (equal prior quadratic classifier) | Imminent apnoea (within a predefined time window) | Continuous recording | 5.5 minutes prior until apnoea event | Cross-validation (40 fold) | Average across patients  (Significant in 3/6 infants)  IBI AUROC 0.72  RRI AUROC 0.67  IBI,RRI AUROC 0.73 | Research |
| **Williamson et al. (2013) (118)** | Apnoea prediction | Preterm infants (25.4-31 weeks GA, 0.78-1.5kg BW) | Not specified | 6 infants  (single centre) | IBIs, heartbeat intervals, movement feature | Retrospective | Machine learning (Gaussian Mixture Model) | Imminent apnoea (within a predefined time window) | Continuous recording | 5.5 minutes prior until apnoea event | Cross-validation (40 fold) | Average across patients:  (Significant in 5/6 infants)  RRI + movement AUROC 0.77  RRI,IBI+movement AUROC 0.75  Combined AUROC 0.80 | Research |
| **Shirwaikar et al. (2016) (115)** | Apnoea prediction | Newborns (including term and preterm infants) | Not specified | 229 apnoea samples (number of infants not specified) | 14 predictors, including continuous 72 hour average heart rate data | Retrospective | Multiple models including decision tree (C5.0), Support Vector Machine (SVM) and ensemble approach (including random forest) – random forest was highest performing | Risk of apnoea during first week of NICU stay | At birth or at NICU admission | First week of NICU admission | Internal validation (holdout dataset) | Random forest classifier:  AUROC 0.82  Accuracy 0.88  Kappa 0.72 | Research |
| **Zuzarte et al. (2021) (119)** | Apnoea prediction | Preterm infants  (27.3-33.9 weeks GA, 29.4 – 34.3 weeks PMA) | Hydrocephalus, IVH > grade 2, congenital defects or bronchopulmonary disease | 10 infants (single centre) | Interbreath intervals, heartbeat intervals, movement features | Retrospective | Unsupervised Gaussian Mixture Model;  Supervised  Logistic regression | Imminent apnoea, bradycardia and hypoxia | Continuous recording predictions | 15 seconds prior until apnoea event | Cross-validation (stratified k fold) | Average across all infants:  AUROC 0.77  (Inclusion of movement features significantly improved model performance) | Research |
| **Xu et al. (2024) (121)** | Apnoea prediction | Preterm infants  Case group: diagnosis of apnoea (respiratory arrest time > 20 s, a slow heart rate [< 100 beats/min], or a decrease in oxygen saturation)  Control group: no diagnosis of apnoea | Severe congenital malformations, inherited metabolic diseases, critical illness, death within 48 h of admission, transfer to another hospital or abandoned hospitalization, incomplete information | 486 infants - 162 case and 324 control (single centre) | GA at birth, birth length, APGAR at 10 minutes, presence of RDS | Retrospective | Combined (Least Absolute Shrinkage and Selection Operator (LASSO) multivariate logistic regression) | Risk of developing apnoea during NICU stay | At birth/at time of neonatal unit admission | Not specified  (predicts the risk of developing apnea at any point during the NICU stay | Internal validation (holdout dataset) | Sensitivity 82.09%  Specificity 70.47%  Positive predictive value 59.46%  Negative predictive value 88.18%  AUROC 0.831  Accuracy 74.48%  95% confidence limits 0.829  Probability range (decision curve) 0.07-0.1  Validated model:  Sensitivity 67.86%  Specificity 95.71%  Positive predictive value 86.36%  Negative predictive value 88.16% | Research |
| **Chen et al. (2026) (123)** | Apnoea instability prediction | Preterm infants <37 weeks | Infants receiving invasive mechanical ventilation, antibiotics, opioids, morphine or fentanyl at the time of the apnoea. | 146 infants (single centre) | Clinical/demographic features including sex, GA, PMA, weight z-score, respiratory support, APGAR score and dynamic features (heart rate, respiratory rate, saturations, apnoea duration, time since apnoea, 5-minute apnoea rate) | Retrospective | Machine learning (XGBoost) | Risk of apnoea-related cardiorespiratory instability ( defined as: bradycardia (>30% reduction in heart rate) and/or oxygen desaturation (SpO₂ <85%) following an apnoeic event) | Continuous recording | 90 seconds prior until apnoea event  (including post-event period 10 seconds after apnoea cessation) | Internal validation (hold-out dataset) and cross-validation (10-fold) | Clinical/demographic features only:  Out of fold train accuracy 62.8%  Balanced test accuracy 66%  AUROC 0.72 (heldout dataset)  Combined clinical/demographic and dynamic features:  Out of fold train accuracy 75.6%  Balanced test accuracy 75.8%  AUROC 0.83 (heldout dataset) | Research |
